# Health Systems Research in a Complex and Rapidly Changing Context: Ethical Implications of Major Health Systems Change at Scale

**DOI:** 10.1111/dewb.12115

**Published:** 2016-03-09

**Authors:** Hayley MacGregor, Gerald Bloom

**Keywords:** developing world, research ethics, reflexivity, deliberative processes, implementation research, complexity

## Abstract

This paper discusses health policy and systems research in complex and rapidly changing contexts. It focuses on ethical issues at stake for researchers working with government policy makers to provide evidence to inform major health systems change at scale, particularly when the dynamic nature of the context and ongoing challenges to the health system can result in unpredictable outcomes. We focus on situations where ‘country ownership’ of HSR is relatively well established and where there is significant involvement of local researchers and close ties and relationships with policy makers are often present. We frame our discussion around two country case studies with which we are familiar, namely China and South Africa and discuss the implications for conducting ‘embedded’ research. We suggest that reflexivity is an important concept for health system researchers who need to think carefully about positionality and their normative stance and to use such reflection to ensure that they can negotiate to retain autonomy, whilst also contributing evidence for health system change. A research process informed by the notion of reflexive practice and iterative learning will require a longitudinal review at key points in the research timeline. Such review should include the convening of a deliberative process and should involve a range of stakeholders, including those most likely to be affected by the intended and unintended consequences of change.

## Introduction

This paper discusses health systems and policy research in complex and rapidly changing contexts and focuses on ethical issues at stake for researchers working with government policy makers to provide evidence to inform major health systems change at scale.

There is a considerable body of literature on the ethics of clinical research in low‐ and middle‐income country (LMIC) contexts, in particular the ethical issues associated with large international clinical trials directed and funded by external sources. Scholars have focused on areas such as the implications of different socio‐cultural and religious settings for the extension of formalised procedures in the ethics of clinical research to LMIC contexts, including the need to take account of the multiplicity of views of what constitutes ethical practice in different localities, and the processes whereby ethical norms come to be constructed in practice and in institutional settings.^1^ The influence of colonial histories and political economy in shaping how people might respond to externally‐driven medical interventions has also received attention^2^ and the ethnography of medical research is well established as a field of study.^3^ Related scholarship has further underscored issues that arise from doing clinical research in countries characterised by a high burden of disease, health inequalities and poor healthcare provision.^4^ Detailed attention has been given to the implications of such realities for ethics review boards appraising clinical research proposed in LMIC, for example considering whether the views of those recruited into research will be elicited with respect to issues such as adequate consent procedures, risks and benefits of participation, and the cessation of health‐related services at the end of trials.^5^ Suggestions to address these issues in research projects have included the institution of community engagement mechanisms such as community advisory boards^6^, or the use of rapid ethnographic methods for assessing the views of involved communities^7^, and mechanisms for sharing research results.^8^ Hyder et al^9^ have also emphasised the value of assessing the national and regional health system functioning in deliberations on standards of care in clinical trials.

An in depth review of the literature on international clinical research is not the focus of this paper. However, we recognise the relevance of insights regarding the nature of social relations generated by health research and the dynamics that can attend these engagements when inequalities of power and resources exist.^10^ London^11^ argues that the thorny nature of the issues at stake with clinical research in LMICs has encouraged ethics boards to focus largely on the implications of context for the procedural elements of review, at the expense of considering the broader ethical issues at stake that are connected to the perpetuation of global health inequalities. He argues for a Human Development approach to ethics of health research in LMICs. This chimes with scholarship that explores the principles for public health ethics in an era of global health research.^12^ Pratt and Loff^13^ draw on political philosophy to propose a justice framework to guide review of international clinical research. The importance of the political environment and the social and political structures and levels of social accountability in setting health systems norms and policy values has also been highlighted.^14^

More recently, attention has been drawn to the rise of health policy and systems research (HPSR) as a field and a recognition that the nature of this kind of research, which includes implementation research, brings a distinctive set of ethical issues, specifically also in LMIC contexts^15^ and in conflict settings with weak health systems and significant external intervention from donor agencies and researchers that introduce significant power dynamics.^16^ Krubiner and Hyder^17^ review the differences between HSR and clinical research and outline some of the ethical principles to consider, with a recognition also of the importance of drawing on scholarship in other fields. The concern with external intervention in national contexts has brought to the fore suggestions for the importance of ‘partnership’ as an ethical model.^18^ Pratt and Hyder^19^ use the principle of justice as a lens to unpack the issue of ‘responsiveness’ for HSR in the light of externally driven research. They identify responsiveness to country priorities and country needs as an important principle and suggest in conclusion that consultation with key stakeholders in national governments and attention to ensuring ‘country ownership’ is a key to ethical HSR. They suggest this ‘embeddedness’ as a way to begin to address the power dynamics of health research in LMICs.

It is this suggestion of ‘embeddedness’ as an important principle that we wish to take as our departure point in this paper. In particular, we focus on situations where ‘country ownership’ of HPSR is relatively well established. Some of this research might still have external funding sources, but we are interested here in situations where there is significant involvement of local researchers and usually close ties with policy makers, or indeed even a blurring of boundaries in professional affiliations. The focus is less on external power relations with funders and outside researchers but on internal relations between different kinds of stakeholders, such as between government officials and researchers. We frame our discussion around two country case studies with which we are familiar, namely China and South Africa. Whilst there are many differences, these countries both have a well‐established national research community and research councils, and are confronting significant health service delivery challenges. This situation puts pressure on researchers to provide evidence to inform rapid health systems change and improve access to services. We argue also that it is significant that in both these countries there is a degree of entitlement to heath care from the state. Indeed in South Africa, health has been made a constitutional right.

We begin with discussion of the nature of the research environment in such contexts and the kinds of demands this places on health system researchers to generate evidence to inform change. Our key concern is to consider how health system researchers can produce knowledge of relevance to policy makers in an ethical way. We do not focus on current review procedures in these settings. Rather, we draw out the implications for doing research in such environments in terms of negotiating the political nature of health system priority‐setting and the different moral framings of health problems and responses. We underscore the complex relationships between researchers and government decision‐makers. We then discuss the ethical challenges that arise for researchers given this imperative to be responsive to health system priorities, especially with respect to negotiating positionality and autonomy. The efforts of researchers in such situations are closely linked to broader national development agendas and aspirations, including addressing persistent societal inequalities. Our perspective on these issues is drawn from our location in development studies. We suggest that the concept of reflexivity and the ability to think carefully about one's positionality is important for health system researchers who need to consider how to retain autonomy in research, whilst contributing evidence for health system change. A research process informed by the notion of reflexive practice and iterative learning will require a longitudinal review at key points in the research timeline. Such review should include the convening of a deliberative process and should involve a range of stakeholders, including those most likely to be affected by the intended and unintended consequences of change.

## Complex and rapidly changing contexts: understanding the health system and the research environment

This section describes the research environment that health system researchers face in countries that are experiencing rapid and interconnected changes and where the institutional arrangements within which the health sector is embedded remain relatively weak^20^. These researchers can be viewed as actors, who play a specific role within their health system. We then use the examples of China and South Africa to illustrate the nature of the research environment in such contexts and the implications for the research process and the role of researchers.

### The research environment

The macroeconomic context in the countries referred to above is of an increasing reliance on markets, which are becoming integrated into the global economy, and of rapid urbanisation. This has led to changing patterns of inequality and social exclusion. Population ageing and several factors that increase the risk of chronic non‐communicable diseases, as well as chronic illness from HIV infection, has led to big changes in the pattern of ill health. New technologies are opening up opportunities for managing illness and also bringing new challenges. Ecological changes, such as intensification of animal husbandry, exposure to environmental pollutants and climate change, are introducing populations to new health risks. Health systems previously designed to meet the needs of a poor and largely rural population at low cost and using very basic technology need to change substantially to meet these challenges.

Many countries have pluralistic health systems with a variety of providers in terms of their training, their relationship to government and markets and the degree to which they are regulated. The poor tend to obtain a large proportion of medical advice, services and drugs from health service providers and drug sellers working outside the regulatory framework. Actors within these systems respond to a number of factors, in addition to government policy. These include financial incentives, reputation and widely accepted behavioural norms. Meanwhile, almost universal coverage by the mass media and growing use of mobile phones and social media mean that the population has access to many sources of information and influencing messages beyond those authorised by the state. In many countries, political systems have emerged with competitive elections and the growing influence of a variety of civil society groups. Some health system analysts have likened health sectors with a large number of actors responding to a variety of influences to a complex adaptive system, in which the response to an intervention is strongly affected by the relationships between actors and a variety of known and unknown influences^21^ and power relations.^22^

Another way to understand this situation is through the lens of historical institutionalism, which emphasises the role of institutions, and the formal and informal rules that underpin them, for the effective performance of a system.^23^ These rules include ‘ethical’ norms of behaviour, which make an individual's response to a situation more predictable. One of the major challenges of managing health system development is to build these institutions and construct these norms, including with respect to expectations of the behaviour of health professionals and the nature of care.

The research to generate evidence that can inform change and assist policy‐makers in these complex contexts is likely to be characterised by several features – such as close relationships between research and policy‐makers and even blurring of positions ‐ that are determined by this more complex and unpredictable research environment. We will return to these in the discussion in section 2.4. The following examples from China and South Africa illustrate this environment and the kind of demands that policy‐makers responsible for managing health system change make on researchers.

### China

Over the past three decades, the Chinese health system has had to adapt to a number of inter‐related economic, social and demographic changes associated with that country's transition from a command economy to a socialist market economy.^24^ The health sector has implemented a sequence of reforms to the organisation, management and finance of health services and it is still evolving new norms of behaviour to guide relationships between clients, health workers and other health system actors. This has taken place in response to problems, as they have emerged. The outcome of any one intervention has been unpredictable and it has been impossible to predict the ultimate shape of the health system. Health system managers and policy‐makers have been under constant pressure to manage a process of change; business as usual has not been an option. They have sought strategies to adapt to changing circumstances, whilst minimising the risk of deleterious outcomes.

The government's approach towards managing economic and social change has been to define broad development objectives and encourage local governments to test new approaches for achieving them.^25^ When policy‐makers have anticipated the need to address a new problem or launch a new intervention, they have encouraged a few localities to experiment with new approaches first. They have used lessons from these experiments to inform national strategies. Over time, specialised research institutes (universities and government think tanks) have played a growing role. The management of health sector change has followed this pattern and has involved a growing number of researchers.

During the initial phase of China's economic reforms starting in the late 1970s the major emphasis of national policy was on encouraging economic growth, managing the transition from a command to a market economy and avoiding social disorder. Chinese government revenue fell substantially during the initial phases of transition and government subsidies to public health facilities did not keep up with rising prices. These facilities had to rely on charges to patients to remain financially viable.^26^ At the same time, a large proportion of health workers in rural facilities lacked sufficient training as a result of human resource policies during the 1970s, which downplayed the importance of specialised technical qualifications. This affected the quality of services. During that time, the government priority for its health facilities was similar to that of other sectors: to prevent bankruptcy and mass layoffs of personnel, whilst encouraging these facilities to generate revenue from the provision of services. These objectives were largely achieved, but a number of problems emerged in terms of a rapid rise in health care costs and growing barriers to access to care by the poor.^27^

Zhang et al^28^ report on the role of research during the 1990s in bringing to the attention of policy‐makers the growing problems with the rural health services. Many journal articles and reports documented the rising cost of medical care and the impact on access to services by poor rural residents. The media began to report these findings. In 2005 a major report by the Development Research Centre of the State Council, a high level government think tank, summarised these finding. This report highlighted shortcomings in the health system and opened a policy debate, which eventually led to a big health sector reform in 2009. Research institutes were also instrumental in the design of experimental rural health insurance schemes aimed at reducing the financial barriers to care. These schemes were undertaken in partnership with county governments. They tended to cover large numbers of people and affected many health sector employees. The researchers who undertook these activities were mostly based in universities or government think tanks. They tended to be funded by the Ministry of Health and a number of senior researchers served on government advisory committees. In that position they acted as advocates for increased government funding of rural health services, on behalf of the Ministry of Health.

During the early 2000s there was a substantial change in government policy in favour of ensuring greater inclusion of all population groups. The large body of research and experimentation meant that the Ministry of Health was well placed to advocate for a national rural health insurance scheme, which was launched in 2003. These schemes have received increasing levels of finance since the launch of a major health reform in 2009. There is now a large and growing body of research on the emerging problems within these schemes and on possible strategies for further reform.^29^

The major influences on the design and implementation of local experimental interventions and national strategies are largely political. The change in government policy, described above, was instrumental in the decision to commit government funds to rural health insurance. However, the large body of evidence on the problems with the health system and the demonstration of the feasibility of rural health insurance in the small‐scale experiments meant that the Ministry of Health could act quickly when overall development priorities changed.

This history demonstrates the growing importance of researchers to China's health policy process. Their choice of subjects for investigation and of policy interventions to test can materially affect the direction of health system development. This makes the normative stance they take and the degree to which they seek the perspectives of different stakeholders, including the poor, important. It highlights the need for more thought about the benefits and risks associated with partnerships with local governments to undertake experimental interventions. It also gives importance to the capacity of researchers to design and undertake studies appropriate to a complex context. The training of Chinese researchers has tended to focus on building certain technical skills. For example, China has an active network aimed at strengthening national capacity for health economics research. Chinese centres of health system research are at an early stage in creating mechanisms for ethical review of their work that takes into account their role as significant partners in change management processes.

### South Africa

In South Africa, the research environment is similarly complex and politically influenced, which gives rise to situations for health systems researchers that generate ethical questions that do not have straightforward responses. An interesting case to unpack relates to the high HIV prevalence in the country, an example which illustrates the implications of contested framings of a problem and priorities for research and policy responses. Unfortunate timing meant that the HIV epidemic in South Africa came to the fore at the same time as the country was facing a politically demanding democratic transition in the 1990s. The second elected president of the democratic South Africa, Thabo Mbeki, notoriously denied the connection between the supposed immunodeficiency syndrome and a specified virus, citing poverty as the predominant cause of the evident ill health of a large number of people. In his analysis of the situation, he aligned himself with dissident AIDS scientists, and pointed to a colonial legacy of racism which in his view also biased scientists to conclude that Africans were responsible for the human emergence and spread of diseases such as HIV. The then minister of health subscribed to the same views and refused to invest in a national ART treatment programme, preferring to promote ‘indigenous’ remedies such as African potato and the vitamins sold by the AIDS dissident Matthias Rath.^30^ Some members of the public health establishment at the time also expressed concerns about whether the state could support the cost of a national HIV treatment programme. Furthermore, doubts surfaced as to whether those most affected in very socio‐economically deprived areas would adhere to long term medication. However, in 2001 the NGO Médecins Sans Frontières (MSF) started providing independent HIV treatment in the socio‐economically deprived area of Khayelitsha on humanitarian grounds, and supported research at the site focused on the realities of provision in such environments. Activists and biomedical researchers formed alliances to promote advocacy for universal treatment in South Africa, adopting a position framed in terms of the constitutional right to health and the moral responsibility of the state to act. A research agenda was driven forward on issues such as the long term cost effectiveness in public health terms of treating to prevent progression to AIDS. By 2003 the state had bowed to pressure from activists to establish a free treatment programme. In 2004 the early results of research in the MSF clinic in Khayelitsha suggested better adherence rates than had been anticipated and later research also showed remarkable outcomes for seriously ill people, who had been placed on treatment.^31^

This period of defining reaction to the HIV epidemic in the late 1990s and early 2000s in South Africa illustrates a situation where contestation existed as to the moral framing of the health crisis and its aetiology, and therefore of the best response and policy solutions. It also illustrates the complex alliances and blurred boundaries that existed between advocacy, public health and health systems research, and government policy‐making. In the early 2000s some researchers felt opposed to the government's ideological position on HIV and would not accept the government's lead on priorities. The blurred boundary between political activism and health research had already existed as part of the resistance to the Apartheid state's policies prior to the democratic transition of 1994, and a recent historical precedent thus existed of not trusting the government's health care ideologies or priority‐setting and of researchers actively promoting alternative agendas. It was clear that no one ‘truth’ prevailed in the late 1990s as to how to perceive the ethical issues at stake in the context of the HIV epidemic. Different framings included Mbeki's assertion of a racist conspiracy, whilst researchers and activists framed the lack of government intervention and treatment provision in moral terms, with the Treatment Action Campaign even accusing the Minister of Health, Shabalala‐Msimang, of homicide. The Treatment Action campaign was also condemnatory of the ethics of large multi‐national drug companies and the cost of life‐saving anti‐retroviral therapy. Clinical research also came under increasing ethical scrutiny as certain trials were accused of using African participants as ‘guinea pigs’, promoting externally driven agendas and not sharing the benefits of research equally with African populations.^32^

As the government position on HIV gradually shifted in the 2000s, greater convergence of opinion occurred on priorities for response, with increasing attention to emerging research on how best to respond to HIV in endemic settings characterised by poor populations, and a health system already under pressure. A new Minister of Health in 2008 significantly opened up alliances with civil society, and when Aaron Motsoaledi took up the position in 2009, he instituted campaigns for HIV testing and shaped policies around international evidence for expanding treatment as prevention. In some instances, public health researchers and erstwhile activists have shifted into government policy positions or work closely with government so that this blurring of boundaries has continued. Departments of Health at provincial and national levels look to health systems researchers to give warning of emergent problems as South Africa has been establishing one of the most extensive public ART treatment programmes globally. The significant successes of recent years have been demonstrated in research findings, such as levels of HIV mortality equivalent to comparable populations in the US.^33^ However, concern has also arisen regarding falls in percentages of HIV positive patients retained in care.^34^ This has led policy makers to explore new models of care to promote adherence in treatment programmes, including paying attention to experimentation with ART clubs and community based health workers by NGOs.^35^ The problem of co‐infection with drug resistant TB has been highlighted as MSF continues to experiment and share findings for forms of community‐based treatment. Researchers who feel strongly that certain groups have less access to HIV services or face rights violations, have framed research around giving voice to constituencies of patients as marginalised, such as HIV positive women, or LGBT communities.^36^ Gender activists engaged in public health research have similarly highlighted their view that integration of Sexual and Reproductive Health with HIV services requires more attention.^37^

With increasing recognition of a burden of non‐communicable disease in the South Africa, attention has shifted to the lessons that can be learned from experience with HIV programmes in order to make health systems more able to provide care and support for all chronic lifelong conditions. Some frame this in terms of justice and ensuring equity by extending similar levels of care and sharing considerable donor and state resources that have been invested in HIV over the past decade. Given resource limitations and some resistance from dedicated HIV specialists to reducing budgets for HIV or harmonising care for chronic lifelong conditions, and concerns about the possible unintended consequences on HIV indicators, the political nature of framings and responses is again very evident as ‘integration’ becomes a disputed policy idea. In the current political environment, health systems researchers are indeed expected by government policy makers to provide evidence for action on issues perceived to be the pressing priorities. Gilson and McIntyre^38^ have begun important analysis of some of the gains and practical challenges of working at this interface for health system researchers in South Africa.

### Ethical challenges for research in complex and dynamic contexts

The examples of China and South Africa highlight how the roles of their health system researchers differ in important respects from those undertaking clinical trials and other medical experiments. Whilst we have not studied ethics committees in the countries, we have drawn in the examples on general observations from our experience. In this section we discuss some features of research that we see as important in order to appreciate the ethical tensions and the challenges for HPSR in such complex and dynamic settings. We have focused on researchers involved in HPSR in close relationship with national policy makers and our observation is that this kind of relationship is increasingly important and needs to be taken into account. Our case examples illustrate how researchers are involved in experimental interventions, which can rapidly lead to the implementation of reforms at scale.

Krubiner and Hyder^39^ propose a framework for understanding the ethical issues of HSR that incorporates ‘systems thinking’. This involves an understanding of the way in which such research can catalyse change at the level of the health system and have consequences and impact on an extensive scale. Similarly, in the complex research environments that we have discussed, the interventions associated with HPSR are much less circumscribed and controlled than the archetypal study for which medical ethics has evolved and uncertainty is inevitable. Often, whole populations and communities are affected as opposed to individual participants in a drug trial. It would be a challenge to ensure that all those potentially affected by the intervention could agree to it, or to ensure that individuals could withdraw. In many cases, the timeframe of the intervention can be long and involve significant engagement with a particular community and locality over a long period. It is difficult to differentiate between the ethical considerations of the initial experimental interventions and those concerning policy reform.

Issues of unequal power relationships are important and are expressed through disputed framings of the problems to be addressed as well as the appropriate interventions. If the viewpoints of the poor and socially excluded are not taken into account, the interventions are unlikely to meet their needs. Institutions are often frail, making both the short and long‐term impact of an intervention unpredictable. One characteristic of this kind of complex reality is that there is a great deal of uncertainty about the outcome of an intervention.

In this context, where government officials are implementing complex change processes, the boundary between a researcher, a government official and a member of an organisation advocating major reforms can be blurred so that the view of the researcher and her study as outside the health system is not tenable. This makes role boundaries harder to define as the researcher becomes a player in a complex environment. Perturbing the environment is likely to have consequences, some of which are unintended. The researcher operates in a reality where agency is diffused and policy processes are political and contested.

In this reality, the work of the typical ethical review board is unlikely to address all the ethical challenges that researchers engaged in health system change processes face. The following section outlines concepts that we see as important for underpinning ethical research and for considering how to adapt and support ethical review in these contexts.

## Implications for the ethical conduct and review of health systems research in such settings

In South Africa and China, researchers have well‐established roles in helping the health system adapt to rapidly changing realities. In both countries they have privileged access to information and their outputs are perceived as having legitimacy and reflecting aspects of the real situation. This legitimacy derives from the reputation of universities as centres of scientific excellence and also from the use to which research findings have been put. In South Africa this legitimacy derives, in part, from the role of researchers in documenting major health system problems during the Apartheid period and in China it reflects the growing use of research findings by government officials in policy debates. In both countries, the researchers have some autonomy in defining research topics and in producing and publishing findings. This autonomy is underpinned by a level of security of employment. Their decisions can influence policy debates. This raises questions about how they should use this power ethically. We propose some responses to these questions below.

### Make normative position clear and encourage reflexivity

In the selection of research topics and in drawing inferences from the findings it is important to make the normative position of the researcher clear and to encourage researchers to reflect on the implications of their positions and roles for the research. In both South Africa and China the government has stated a commitment to the provision of universal access to high priority health services. In both cases, the research discussed took this as an overall objective. Whilst responsiveness to national research priorities is an important principle, the position of researchers might not always align with some or all government policy‐makers. We suggest that it is important to convene dialogue with policy‐makers and other stakeholders during research proposal development and that this dialogue should include discussion of normative positions. This will require reflection on the part of researchers as to their positionality in order to negotiate and retain autonomy. One strategy could be to accept the blurred boundaries between researchers and policy‐makers if these are present, as well as the positioning of researchers as partly on the ‘inside’. An aspect of the research might include monitoring of change due to an intervention and explicit analysis of the effects of such change and the implications. It might be appropriate to conceptualise the role of the health system researchers as being part of a process of change, whilst also evaluating change and observing for unintended consequences. Such a positioning of researchers as engaged in ‘learning by doing’ in the research process, rather than taking up the role of examining as outside observers, would mark a clear departure. An ability on the part of researchers to be reflexive will also increase awareness of the situations where it might be necessary to negotiate with policy makers to ensure independence in the research focus and analysis in order to retain autonomy. An ongoing regular dialogue with policy‐makers throughout the duration of research and attention to those relationships and clear channels of communication might well make such negotiation easier.

If we take the normative position that policy should benefit the poor and socially excluded, it will be important to assess intended and unintended consequences of a policy initiative for them. In a complex context, it is impossible to predict the outcome of an intervention and it is important to assess multiple points of view. It is especially important to take into account the perspectives of the poor and less powerful.

### Define the kind of evidence necessary to inform decision‐making

The design of health systems research needs to take into account the context and the likely responses of the different actors.^40^ It should also take into account the need for systematic information on the functioning and organisation of the system before the implementation of an intervention at scale. The ability to undertake this kind of scoping work is a core skill for those engaged in HSR. One aspect of ethical review is to ensure that an intervention is based on this kind of analysis. The justification for a specific study and of the costs of the study financially and in terms of risks and inconvenience to the population, needs to be in terms of an appreciation of the knowledge needs for effective change management. This includes systematic knowledge about the likely response of the health system when findings of research are applied at scale so that policy inferences can be drawn.

### Ensure representative partnerships and convene a deliberative process

We have outlined how health systems in complex and dynamic contexts involve many actors, in both the formal and informal sectors. We argue that it is important to understand this plurality and attempt to ensure representation of different stakeholders in discussion about the framing and ethics of research and of proposed interventions. In the first instance, this involves dialogue and forms of partnership with state policy‐makers and potentially also with relevant bureaucrats or senior managers in the health system. However, consultation needs to extend beyond government actors to extent to civil society actors and beyond.

With respect to the views of the less powerful, one way to ensure that the points of view of local people are considered is to establish some form of community engagement as a part of health systems research, as has been suggested for clinical research. This is not a simple process. London's^41^ comments on the dangers of assuming communities are homogeneous, or neglecting the agency of marginalised people with respect to seeking health, remain very pertinent. Health system research involving the implementation of health systems change at scale has the potential to provide major benefits to a community or population. However, this kind of research, depending on the nature of the change, can also entail risks, especially to the poor and relatively powerless. They are vulnerable to potential unintended consequences of a change process, which could, for example, reduce access to services or impair their quality. The community affected by such change might have a different perception of these risks or the consequences compared to the researchers. In a similar vein, it is important to appreciate how large scale health system change inevitably intersects with diverse social and political aspirations and understandings of what constitutes desirable change for the public good and how to achieve this. This involves an understanding of broader societal views of harm, and not just harm to individuals in medical interactions. The views of those who negotiate a health system and make do in the face of constraints to achieving good health are crucial.

It is also difficult to ensure that all those potentially affected are fully informed of all possible beneficial and deleterious outcomes of an intervention. In each case, it is important to determine the processes that constitute consent to undertake an intervention. It is also important to define the responsibilities of the researcher to monitor possible negative outcomes, especially for the poor and powerless and ensure that action is taken to mitigate these harms. We suggest that it is important for researchers to convene deliberative processes that involve a range of stakeholders prior to the initiation of research and at key points in the research timetable.

### Accept and address uncertainty

Even a well organised ethical review cannot adequately ensure that unnecessary suffering is avoided, especially in complex and rapidly changing contexts. It is important to monitor for potentially deleterious unintended outcomes and arrangements should be made to modify implementation of the research on the basis of findings. This will involve a review of design and the potential ethical impact at regular intervals. This requires the researchers to build an iterative mechanism into the research process for careful monitoring of outcomes and identifying consequences and risks. This iterative review process should occur at regular intervals in the timeframe of the research and the researchers should decide carefully what information should be brought to the table for such review. We recognise that ongoing monitoring of outcomes and risks would be burdensome if instituted for all HSPR. Consideration could be given to specifying for review boards the kinds of research or the research contexts where this would seem particularly necessary, such as when an intervention is proposed that is assessed as carrying a significant risk of potential unintended consequences.

### Consider and address the timeframe of research

Timescale in an important consideration for HSR. If the study concerns the implementation of major changes, it is likely to take a considerable amount of time and raises additional ethical considerations.^42^ These include important questions about the measures to be taken to sustain any interventions or to inform the community if interventions are to be time limited. In such instances, further dialogue is required regarding what disengagement would mean.

## Conclusion

We have discussed the ethical challenges faced by researchers in complex and dynamic contexts where they are working closely with policy‐makers to provide evidence for rapid health systems change at scale. We suggest that longitudinal review should be considered at key points in the HPSR project timeline as opposed to the traditional one off review prior to the inception of research. Guidelines could be developed to identify kinds of HSPR where iterative monitoring would be particularly necessary. We also maintain that a component of ethics review should involve the convening of a deliberative process involving participation by several key stakeholders and pay attention to achieving adequate representation. Such a process could encourage iterative learning and require adaptation on the part of researchers. In contexts where social accountability mechanisms have been established in the health system, these could be engaged alongside civil society organisations. We contend that convening deliberative platforms at key intervals in the research timeline is important in complex contexts. However, reflexivity and critical reflection on the part of researchers is also necessary in approaching such ‘partnership’ with stakeholders in HPSR, and should be underpinned by a commitment to social accountability. As researchers and the institutes that employ them continue to develop their role as important contributors to the management of health system innovation and change in a complex and dynamic context, they will need to establish mechanisms to ensure they work in a competent and ethical manner. Our aim is to stimulate debate about the processes of review and reflection that could contribute to achieving this purpose.

## Notes

1 Z.A. Bhutta. Ethics in International Health Research: A Perspective from the Developing World. *Bull World Health Organ* 2002; 80: 114‐120; I. Harper. 2007. Translating Ethics: Researching Public Health and Medical Practices in Nepal. *Soc Sci Med* 65: 2235‐2247; A. Wahlberg et al. From Global Bioethics to Ethical Governance of Biomedical Research Collaborations. *Soc Sci Med* 2013; 98: 293‐300; P. Kingori. Experiencing Everyday Ethics in Context: Frontline Data Collectors’ Perspectives and Practices of Bioethics. *Soc Sci Med*, 2013; 98: 361‐370. F.N. Chukwuneke et al. Global Bioethics and Culture in a Pluralistic World: How Does Culture Influence Bioethics in Africa? *Ann Med Health Sci Res* 2014; 4: 672‐675.

2 P.W. Geissler. ‘Kachinja are Coming!’ Encounters around a Medical Research Project in a Kenya Village. *Africa*. 2005; 75:173‐202; J. Fairhead, M. Leach et al. Where Techno‐Science Meets Poverty: Medical Research and the Economy of Blood in The Gambia, West Africa. *Soc Sci Med* 2006; 63: 1109‐1120; P.W. Geissler and R. Pool. Popular Concerns with Medical Research Projects in Africa – A Critical Voice in Debates about Overseas Research Ethics. *Trop Med Int Health* 2006; 11: 975–982.

3 S. Molyneux & P.W. Geissler. Ethics and the Ethnography of Medical Research in Africa. *Soc Sci Med* 2008; 67: 685‐695.

4 S.R. Benatar. Reflections and Recommendations on Research Ethics in Developing Countries. *Soc Sci Med* 2002; 54: 1131‐1141; J. Azetsop. New Directions in African Bioethics: Ways of Including Public Health Concerns in the Bioethics Agenda. *Dev World Bioeth* 2011; 11:1 4‐15; T. Lahey. The Ethics of Clinical Research in Low‐ and Middle‐Income Countries. *Handb Clin Neurol* 2013; 118: 301–313.

5 V. Marsh et al. Managing Misaligned Paternity Findings in Research including Sickle Cell Disease Screening in Kenya: ‘Consulting Communities’ to Inform Policy. *Soc Sci Med*, 2013; 96: 192‐199; S. Molyneux et al. Conducting Health‐Related Social Science Research in Low Income Settings: Ethical Dilemmas Faced in Kenya and South Africa. *J Int Dev* 2009; 21: 309–326

6 M. Boga et al. Strengthening the Informed Consent Process in International Health Research through Community Engagement: The KEMRI‐Wellcome Trust Research Programme Experience. *PLoS Med* 2011; 8:9: 1‐4; I. Jao et al. Involving Research Stakeholders in Developing Policy on Sharing Public Health Research Data in Kenya: Views on Fair Process for Informed Consent, Access Oversight, and Community Engagement. *J Empir Res Hum Res Ethics* 2015; 10: 264‐277; V. Marsh, *op.cit*. note 5.

7 A. Addissie et al. A Mixed‐Methods Study on Perceptions Towards Use of Rapid Ethical Assessment to Improve Informed Consent Processes for Health Research in a Low‐Income Setting. *BMC Med Ethics* 2014; 15:35: 2–12.

8 S. Bull et al. Best Practices for Ethical Sharing of Individual‐Level Health Research Data from Low‐and Middle‐income Settings. *J Empir Res Hum Res Ethics* 2015; 10: 302–313.

9 A.A. Hyder & L. Dawson. Defining Standard of Care in the Developing World: the Intersection of International Research Ethics and Health Systems Analysis. *Dev World Bioeth* 2005; 5: 142–152

10 S. Molyneux, D. Kamuya & V. Marsh. Community Members Employed on Research Projects Face Crucial, Often Under‐Recognized, Ethical Dilemmas. *Am J Bioeth* 2010; 10: 24‐26; T. Chantler et al. Ethical Challenges that Arise at the Community Interface of Health Research: Village Reporters’ Experiences in Western Kenya. *Dev World Bioeth* 2013; 13: 30–37.

11 L. London. Ethical Oversight of Public Health Research: Can Rules and IRBs Make a Difference in Developing Countries? *Am J Public Health* 2002; 92: 1079–1084.

12 C. Chakraborti. Systemic Negligence: Why it is Morally Important for Developing World Bioethics. *Dev World Bioeth* 25 April 2014; 1‐6; S. R. Benatar, P.A. Singer & A.S. Daar. Global Challenges: The Need for an Expanded Discourse on Bioethics. *PLoS Med* 2005; 2:7; C.B. IJsselmuiden et al. Evolving Values in Ethics and Global Health Research. *Glob Public Health* 2010; 5: 154–163.

13 B. Pratt & B. Loff. A Framework to Link International Clinical Research to the Promotion of Justice in Global Health. *Bioethics* 2014; 28: 387–396.

14 A.A. Hyder et al. Integrating Ethics, Health Policy and Health Systems in Low‐ and Middle‐Income Countries: Case Studies from Malaysia and Pakistan. *Bull World Health Organ* 2008; 86: 606‐611; G.M.A Hussein. Democracy: the Forgotten Challenge for Bioethics in the Developing Countries. *BMC Med Ethics* 2009; 10:3 1–4.

15 A.A. Hyder et al. (a) The Ethics of Health Systems Research in Low‐ and Middle‐Income Countries: A Call to Action. *Glob Public Health* 2014; 9: 1008‐1022; A.A. Hyder et al. (b) Ethical Review of Health Systems Research in Low‐ and Middle‐Income Countries: A Conceptual Exploration. *Am J Public Health* 2014; 14:28–37.

16 P. Hill. Ethics and Health Systems Research in ‘Post’–Conflict Situations. *Dev World Bioeth* 2004; 4: 139–153.

17 C.B. Krubiner & A.A. Hyder. A Bioethical Framework for Health Systems Activity: A Conceptual Exploration Applying ‘Systems Thinking’ *Health Systems* 2014; 3:124–135

18 D.W. Dowdy. Partnership as an Ethical Model for Medical Research in Developing Countries: the Example of the ‘Implementation Trial’. *J Med Ethics* 2006; 32: 357–360.

19 B. Pratt & A.A. Hyder. Reinterpreting Responsiveness for Health Systems Research in Low and Middle‐Income Countries. *Bioethics* 2015; 29: 379–388.

20 D. Leonard et al. Institutional Solutions to the Asymmetric Information Problem in Health and Development Services for the Poor. *World Dev* 2013; 48: 71–87.

21 D. Peters. The Application of Systems Thinking in Health: Why use Systems Thinking? *Health Res Policy Syst* 2014; 12(1).

22 L. Gilson et al. Advancing the Application of Systems Thinking in Health: South African Examples of a Leadership of Sensemaking for Primary Health Care. *Health Res Policy Sys* 2014; 12 (1).

23 P. Pierson & T. Skocpol. 2002. Historical Institutionalism in Contemporary Political Science. In I. Katznelson & H. Milner eds *Political science: The State of the Discipline* 693–721 New York: Norton.

24 G. Bloom. Building Institutions for an Effective Health System: Lessons from China's Experience with Rural Health Reform. *Soc Sci Med* 2011; 72: 1302–1309.

25 L. Husain. Logics of Government Innovation and Reform Management in China. *STEPS Working Paper* No. 85. Brighton: STEPS Centre 2015.

26 Yip, W. and Hsiao, W. 2015. What Drove the Cycles of Chinese Health System Reforms?, *Health Systems and Reform* 1:1, 52‐61.

27 G. Bloom. Building Institutions for an Effective Health System: Lessons from China's Experience with Rural Health Reform. *Soc Sci Med* 2011; 72: 1302‐1309; W. Yip & W. Hsiao. What Drove the Cycles of Chinese Health System Reforms? *Health Systems and Reform* 2015; 1: 52‐61; Duckett, J. 2011 *The Chinese State's Retreat from Health: Policy and the Politics of Retrenchment* Oxford: Routledge.

28 Z. Zhang, L. Fang & G. Bloom. 2010. The Rural Health Protection System in China in *Health Policy in Transition: The Challenges for China* V. Lin et al. eds Peking University Medical Press.

29 X. Liang et al. The Effect of New Cooperative Medical Scheme on Health Outcomes and Alleviating Catastrophic Health Expenditure in China: A Systematic Review. PLoS ONE 2012; 7(8) e40850.

30 S. Robins & B. von Lieres. Remaking Citizenship, Unmaking Marginalization: The Treatment Action Campaign in Post‐Apartheid South Africa *Can J Afr Stud* 2004; 38:575‐586; D. Fassin & H. Schneider. The Politics of AIDS in South Africa: Beyond the Controversies. *BMJ,* 2013; 326:495‐7; D. Fassin. 2007. *When Bodies Remember: Experiences and Politics of AIDS in South Africa,* Los Angeles: University of California Press; N. Nattrass. 2007. *Mortal Combat: AIDS Denialism and the Struggle for Antiretrovirals in South Africa,* Durban: University of Kwa‐Zulu Natal Press; N. Geffen. Echoes of Lysenko: State‐Sponsored Pseudo‐Science in South Africa. *Soc Dyn* 2005; 31**:** 182–210.

31 D. Coetzee et al. (2004). Promoting adherence to antiretroviral therapy. *AIDS*, 18 (Supplement 3) S27‐S31; A. Boulle, et al. Seven‐Year Experience of a Primary Care Antiretroviral Treatment Programme in Khayelitsha, South Africa. *AIDS* 2010; 24: 563–572.

32 Lahey, *op. cit*. note 4.

33 A. Boulle et al. Mortality in Patients with HIV‐1 Infection Starting Antiretroviral Therapy in South Africa, Europe, or North America: A Collaborative Analysis of Prospective Studies. *PLoS Med* 2014; 11: 1001718.

34 M. Osler et al. A Three‐Tier Framework for Monitoring Antiretroviral Therapy in High HIV Burden Settings. *J Int AIDS Soc* 2014; 17(1).

35 M. Luque‐Fernandez et al. Effectiveness of Patient Adherence Groups as a Model of Care for Stable Patients on Antiretroviral Therapy in Khayelitsha, Cape Town, South Africa. *PLoS ONE* 2013; 8:56088; G. Fatti et al. Improved Survival and Antiretroviral Treatment Outcomes in Adults Receiving Community‐Based Adherence Support: 5‐Year Results From a Multicentre Cohort Study in South Africa. *J Acquir Immune Defic Syndr*, 2012; 61:50‐58.

36 A. Muller & H. MacGregor. Sexual and Reproductive Health Rights of Women Living with HIV in South Africa *IDS Evidence Report 3* Brighton: Institute of Development Studies March 2013.

37 J. Kehler et al. 2010. *Where are the Human Rights for Pregnant Women? Scale‐Up of Provider‐Initiated HIV Testing of Pregnant Women: The South African experience* Cape Town: AIDS Legal Network; S. Cooper & J. Harries, 2009. If I Could Properly Understand and get the Right Information: A Qualitative Study on the Sexual and Reproductive Health Needs and Rights of HIV‐Positive Women, CSSR Working Paper Series, 256.

38 L. Gilson & D. McIntyre. The Interface Between Research and Policy: Experience from South Africa. *Soc Sci Med* 2008; 67: 748–759.

39 Krubiner & Hyder, *op.cit* note 17.

40 G. Bloom & S. Wolcott. 2012. Building Institutions for Health and Health Systems in Contexts of rapid change. *Soc Sci & Med*; 2012. 96:216‐222.

41 London, *op.cit*. note 11.

42 A.A. Hyder et al. Exploring the Ethics of Long‐Term Research Engagement with Communities in Low‐ and Middle‐Income Countries. *Public Health Ethics*. 2012.5:252‐262

